# Hip arthroscope-assisted percutaneous reduction and fixation of displaced subcapital femoral neck fracture

**DOI:** 10.3389/fsurg.2025.1555752

**Published:** 2025-04-01

**Authors:** Dajiang Du, Che Zheng, Mengxin Xue, Jiewei Chen, Yiyang Ma, Yun Gao, Changqing Zhang

**Affiliations:** Department of Orthopedic Surgery, Shanghai Sixth People’s Hospital Affiliated to Shanghai Jiao Tong University School of Medicine, Shanghai, China

**Keywords:** subcapital femoral neck fracture, arthroscopically assisted reduction and internal fixation (ARIF), reduction quality, minimal invasion, case

## Abstract

Reduction quality is associated with fracture prognosis. Displaced subcapital femoral neck fracture has highest possibility of avascular necrosis of femoral head and non-union among the femoral neck fractures, which commonly necessitate revisions or hip replacement. This study introduces for the first time of using hip arthroscope to directly visualize and assist the reduction of displaced subcapital femoral neck fracture when closed reduction is unsatisfactory. Due to the minimally invasive advantage of arthroscopic assistance, radiation exposure or intraoperative bleeding can be reduced, open reduction is avoided so that the blood supply of femoral head can be preserved. Through direct visualization, the complete removal of hematoma and fracture debris can be achieved, which is not possible with closed reduction, and can potentially reduce the risk of non-union during bone healing.

## 1 Introduction

Subcapital femoral neck fracture suffers the worst prognosis among the intracapsular fractures, which is associated with reduction difficulty (1). The incidences of femoral neck non-union and avascular necrosis after displaced femoral neck fractures treated with internal fixation vary between 11%–19% and 10%–30%, respectively (2). Successful reduction helps reduce complications such as avascular necrosis of femoral head and non-union in subcapital femoral neck fracture (2). Although reduction performed under direct visualization is a guarantee of quality, open reduction can cause increased bleeding, significant soft tissue damage and may damage the blood supply to the femoral head (3). On the other hand, hip arthroscopy provides the possibility of precise reduction with minimally invasive advantages.

Satisfactory reduction quality of femoral neck fracture is important for the operation success. Continuous medial and lateral cortical lines without varus angulation in the AP view and continuous anterior and posterior cortical lines without retroversion in the lateral view are considered acceptable reduction quality (4). When closed reduction yields unsatisfactory result, percutaneous and open reduction are usually applied to improve the reduction quality (5). However, due to the soft tissue thickness, the effectiveness of percutaneous reduction is limited. Intraoperative time and bleeding will increase while the blood supply around the joint capsule might be impaired by open reduction (6). Therefore, a minimally invasive technique that can directly visualize the reduction without damaging the blood vessels is needed in order to improve the reduction quality for the irreducibly displaced subcapital femoral neck fracture.

Hip arthroscopically assisted reduction and internal fixation (ARIF) is a minimally invasive procedure and is able to provide direct visualization during fracture reduction. However, due to the long learning curve of hip arthroscopy and the inability to tract the joint space because of the facture in the femoral neck, no study has been reported using conventional arthroscopic technique to assist in the reduction of femoral neck fractures so far. In this study, we are introducing an “outside-in” hip ARIF with slight longitudinal traction to assist the reduction of subcapital femoral neck fracture. Under the direct visualization of the fracture line, hip ARIF reduces the radiation exposure or intraoperative bleeding caused by open reduction. Furthermore, fracture ends can be thoroughly debrided under direct visualization which effectively improves the reduction quality without disrupting the blood supply to the femoral head, thus reducing the chances of avascular necrosis or non-union caused by soft tissue impaction. Lastly, the accurate reduction and compression effect can be supervised directly under hip ARIF.

## 2 Methods

**Figure 1:**
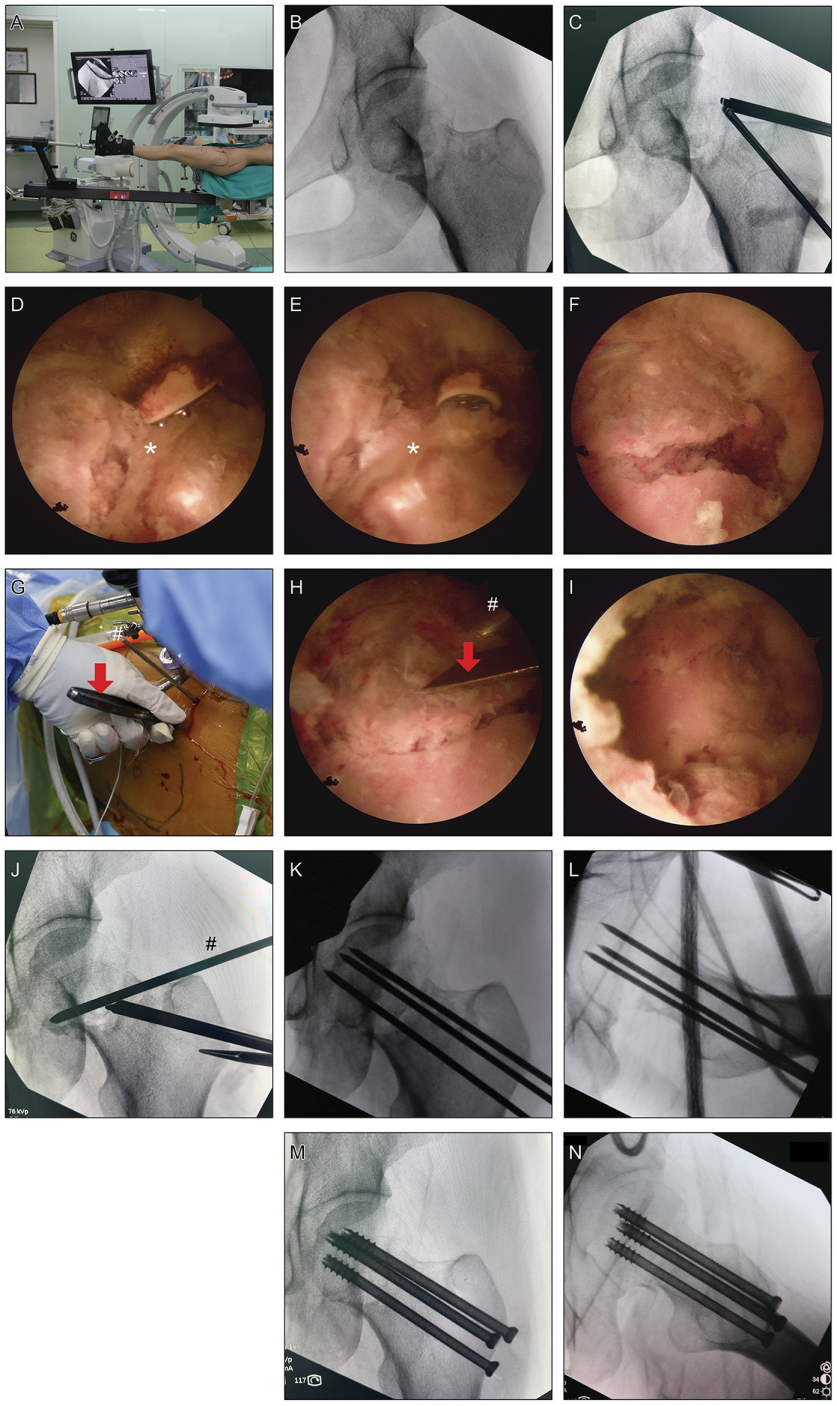
Surgical procedures of hip arthroscopy-assisted percutaneous reduction and fixation of displaced subcapital femoral neck fracture. **(A)** Patient was laid supine on Hana surgical bed with the injured leg positioned in the traction boot. **(B)** Reduction under traction and rotation was attempted yet with unsatisfactory result. **(C)** “Outside-in” technique was used to create anterolateral and lateral portals pointing to the fracture line. **(D–F)** After exposing the fracture line, soft tissues, probably annular ligament, that were caught between the fracture gap were debrided using probe and curette (*, soft tissues between the fracture gap). **(G–I)** A 3.0-mm K-wire was inserted under supervision into the anterolateral part of the femoral head as a joystick to control the orientation of the femoral head and a fine periosteal elevator was inserted through the lateral portal to further assist the reduction (red arrows, periosteal elevator; #, 3.0-mm K-wire). **(J)** According to the reduction effect under supervision, the traction level, internal or external rotation, and adduction or abduction of the surgical leg were coordinately adjusted using the traction boot to yield satisfactory reduction. **(K,L)** Intraoperative fluoroscopy after the insertion of K-wires for provisional fixation. **(M,N)** Intraoperative fluoroscopy after the insertion of three 7.0-mm cannulated cancellous screws.

Patient was laid supine on Hana surgical bed (Mizuho, US) with the left leg positioned in the traction boot (Figure 1A). Reduction under traction and rotation was attempted yet with unsatisfactory result (Figure 1B).

**Figure 2:**
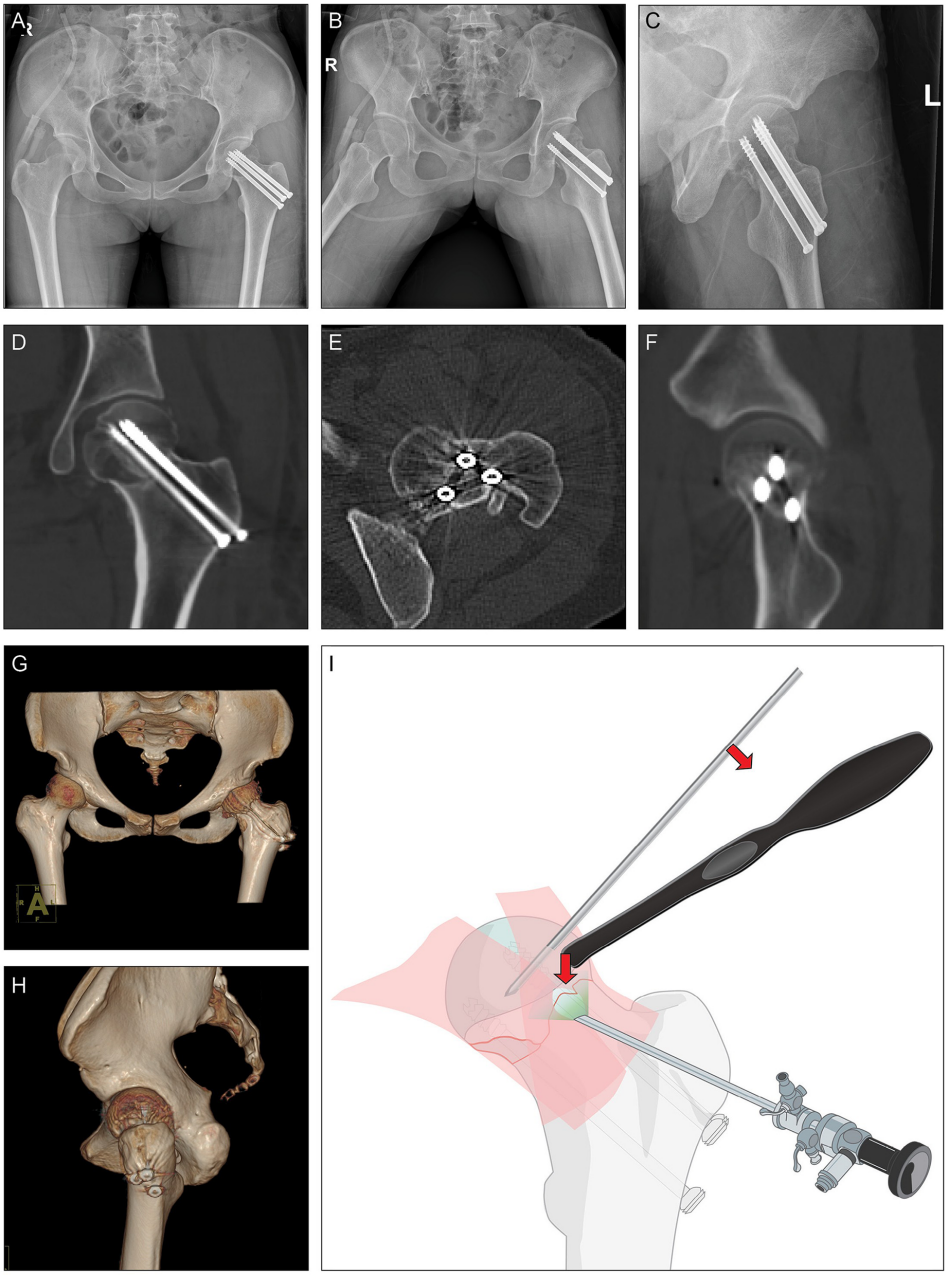
Postoperational radiology. **(A–C)** Postoperational x-rays and **(D–H)** CT scan. **(I)** Illustration of hip arthroscopy-assisted percutaneous reduction and fixation of displaced subcapital femoral neck fractur.

“Outside-in” technique was used to create anterolateral and lateral portals pointing directly to the fracture line with the help of fluoroscopy (7, 8) (Figure 1C). Longitudinal capsulotomy was performed with a COBLATION wand (Smith & Nephew, UK). Care should be taken not to harm the labrum. During capsulotomy, intra-capsular hematoma was debrided thoroughly. After exposing the fracture line, soft tissues that were caught between the fracture gap were further debrided using probe and curette (Figures 1D–F). Next, a 3.0-mm K-wire was inserted under supervision into the anterolateral part of the femoral head as a joystick to control the orientation of the femoral head and a fine periosteal elevator was inserted through a portal to further assist the reduction (Figures 1G–I and Figure 2I). Meanwhile, according to the reduction effect under direct supervision, the traction level, internal or external rotation, and adduction or abduction of the surgical leg were coordinately adjusted using the traction boot.

After yielding satisfactory fracture reduction (Figure 1J), K-wires were inserted through the fracture line for provisional fixation. Reduction quality was then confirmed in AP, lateral and axial views (Figures 1K,L). Three 7.0-mm cannulated cancellous screws (Zimmer, US) over the guide-wires were fixed parallel to the head-neck axis (Figures 1M,N).

## 3 Materials and equipment

4.0 mm hip arthroscopy system with 70-degree 4 K scope and QUANTUM 2 COBLATION system (Smith & Nephew, UK) were used for the direct supervision of percutaneous reduction.

## 4 Case

**Figure 3:**
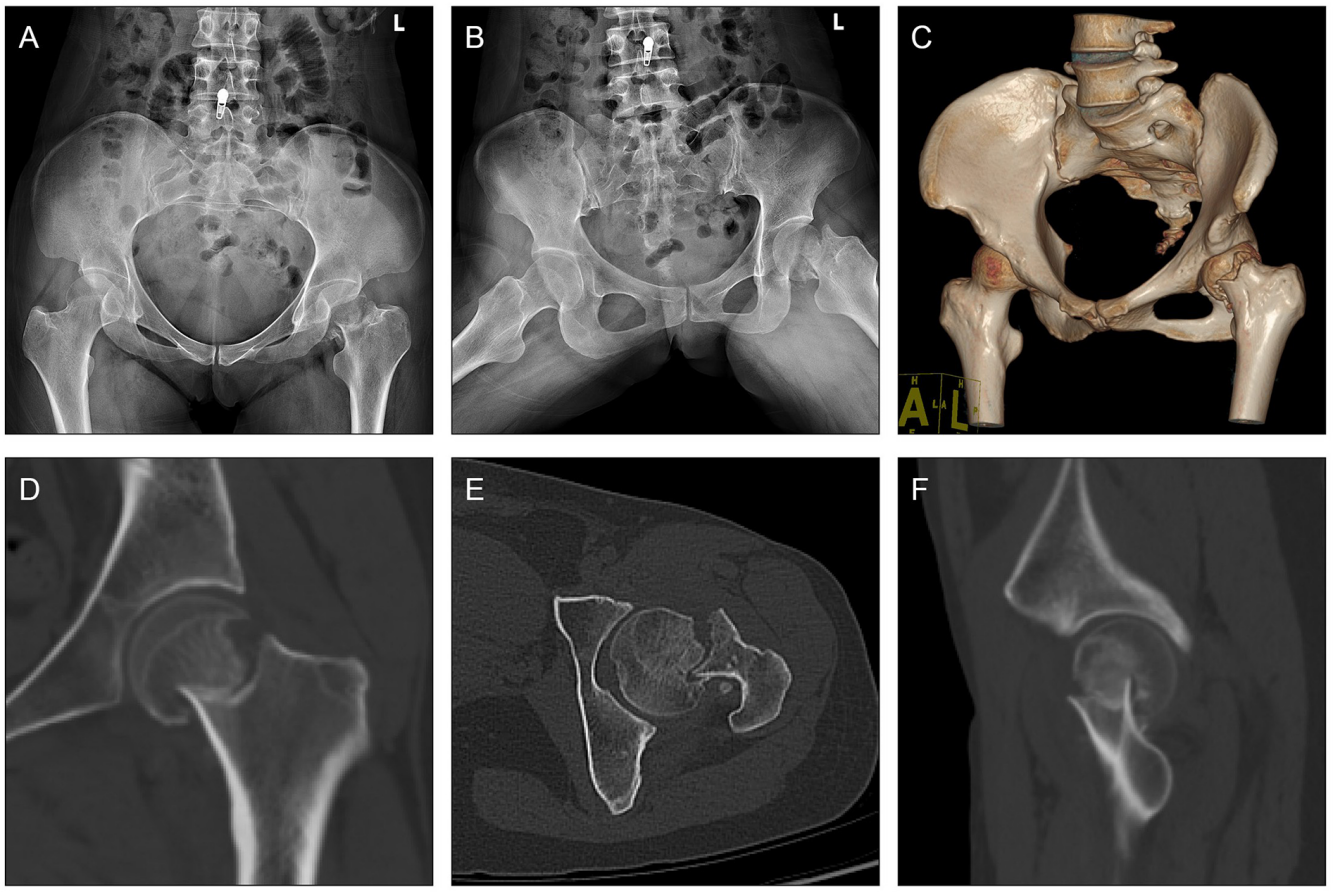
Case presentation. **(A,B)** Pre-operative x-rays and **(C–F)** CT scan of a 36-year-old female who was diagnosed with displaced subcapital femoral neck fracture (AO/OTA classification: 31B1.3, Pauwels classification: Type I) caused by a fall from flat during a sport accident.

Presented case is a 36-year-old female who suffered from displaced subcapital femoral neck fracture (AO/OTA classification: 31B1.3, Pauwels classification: Type I) (Figure 3). The trauma was caused by a fall from flat after a sport accident. The patient had rested at home and tried to walk back on her feet for three days before she went for medical consultancy. The surgery was performed six days after her injury.

After surgery, the patient was allowed for full range of motion while weight bearing of the surgical leg was avoided until post-operative 3rd month.

## 5 Results

With the assistance of hip arthroscope, fracture line was anatomically reduced which was confirmed by x-ray and CT showing continuous cortical lines and trabecular patterns without retroversion or varus angulation in all views (Figure 2). At 1 month after surgery, this patient reported Harris Hip Score of 63 points, Oxford Hip Score of 13 points, EQ-5D of 65 points and VAS pain levels of 2.0 points (Supplementary Table S1). The relatively low hip functional scores can be attributed to the prescribed weight-bearing restrictions during the first three months postoperatively. Notably, the range of motion parameters, including flexion (100–110°), internal/external rotation (25–30°), and abduction (30–40°)/adduction (20–30°) of the hip joint, all approached normal levels (Supplementary Figure S1).

## 6 Discussion

Intracapsular fracture of the femoral neck is associated with avascular necrosis and non-union (9), while high reduction quality is associated with lower complications (10, 11). Closed reduction is often applied with or without the help of percutaneous reduction. When irreducible conditions persist, open reduction becomes inevitable. However, open reduction is associated with higher rates of disruption of blood supply of the femoral head and increased bleeding. Therefore, this study is to introduce a method of using hip arthroscope to assist the reduction of displaced subcapital femoral neck fracture, which avoided open reduction and has effectively improved the reduction quality without disrupting the blood supply to the femoral head.

Basically, arthroscopic assistance is an extension of percutaneous reduction plus direct visualization. Although controversial, scopes have been used during fracture reduction in many scenarios (12, 13). On top of small incisions, the advantageous aspects of an arthroscopic surgery include reducing pain, the blood loss and the risk of complications, yet with speedy recovery. For the first time, we report the use of the “outside-in” technique (7, 8) to position the hip arthroscope on the surface of the joint capsule proximal to the fracture line. The joint capsule was then incised in a limited manner, allowing for the precise reduction of the femoral neck fracture under direct arthroscopic visualization.

Subcapital femoral neck fracture is usually displaced in varus angulation and retroversion, leaving frequent anterior fracture displacement positioned near the annular ligament (2). Such displacement often results in the likelihood of soft tissue impaction at the anterior fracture site during reduction, a situation that is challenging to avoid or to confirm using conventional closed reduction techniques and may lead to subsequent femoral neck non-union (2, 14). Utilizing hip ARIF, we identified and thoroughly excised the impacted soft tissue, presumed to be the annular ligament, that was entrapped between the fracture ends. Such complete debridement allowed for direct bone-to-bone contact at the fracture ends, which is typically difficult to achieve without open reduction.

Furthermore, the femoral heads in the subcapital femoral neck fractures often lack sufficient restraint, resulting in uncontrollable rotation hence irreducibility using conventional closed reduction techniques (14, 15). Although joystick technology facilitates aforementioned difficulty, accurate placement of the K-wire in the femoral head via percutaneous methods can be challenging, often necessitating extensive fluoroscopy and posing risks of iatrogenic injury (16, 17). Hip ARIF enables precise insertion of the joystick into the desired location under direct supervision, thereby enhancing the accuracy and efficiency of the reduction process.

Maintaining the integrity of joint capsule is beneficial for femoral neck fracture reduction (18, 19). Hip arthroscopy requires limited incision of the anterior joint capsule, therefore, accurate fluoroscopic positioning prior to incision is crucial as it prevents excessive capsulectomy that could compromise the restraint provided by the joint capsule. In cases where the distal fracture end sinks or shifts due to the loss of anterior joint capsule support, upward force provided by a joystick or reduction forceps can be applied to the posterior part of the greater trochanter to rectify the displacement. Additionally, excessive localized force during reduction is not advisable, as it may result in iatrogenic fractures. Instead, the use of indirect reduction techniques, such as traction boot manipulation, should be synchronously employed if necessary.

Studies have reported the successful application of arthroscopic treatment of femoral head fractures (20, 21), indicating a potential advantage of intra-articular accessibility of arthroscopic surgery when articular surface requires attention. Techniques and technologies have evolved by bringing with high-resolution cameras mounted in various degrees of scopes. However, in the acute settings involving high energy, comminuted fractures, critically injured patients may not benefit from arthroscopic techniques. While not all fracture types are necessary and suitable for this hip ARIF approach, selective proximal femoral fractures and intracapsular fractures can be systematically evaluated for considering using hip ARIF as an alternative reduction approach. Therefore, the indication of hip ARIF must be established prudently.

One of the technical concerns regarding ARIF is that arthroscopy may increase surgical time. Arthroscopy does require additional setup time, however, which can be done pre-operatively. The total surgical duration performed by an experienced surgeon actually may not increase when compared with open reduction's. A RCT will be performed in future not only to compare the clinical efficacy but also the surgical time between hip ARIF and open reduction.

Increased intracapsular pressure could contribute to the pathogenesis of avascular necrosis (2, 22, 23). So could the hematoma, which can also cause increased intracapsular pressure and is impossible to be debrided by closed reduction. Hip ARIF enables thorough debridement of the hematoma under direct visualization. In addition, the arthroscopic procedure is typically brief and focused, limiting prolonged exposure of the fracture site to relatively higher water pressure. Current literature has not demonstrated a direct causal relationship between arthroscopic irrigation pressure and increased rate of avascular necrosis. However, we recognize that this remains an area requiring further investigation.

However intuitive and direct, mastering the technique of hip arthroscopy has a long learning curve. Unlike the technique introduced previously using hip ARIF to treat femoral head fracture, where it focused on the restoration of articular surface, this case emphasizes the restoration of neck-shaft angle and stability (24). The fundamental principle of both techniques relies on the direct visualization to precise control the reduction through “outside-in” technique. Additionally, joysticks were used to assist in controlling the position of the femoral head, while traction and rotational adjustments were applied to achieve optimal reduction (24). Aforementioned shared technical aspects underscore the versatility and efficacy of hip ARIF in managing different types of hip fractures, while also highlighting the need for tailored strategies based on fracture characteristics and patient-specific factors.

To implement this introduced technique, the surgeon must be proficient in the “outside-in” technique of hip arthroscopy with slight or even without traction, which could limit the dissemination of this technique. The next phase involves conducting studies to design specialized appliances to facilitate this technique. Meanwhile, arthroscopy holds potential limitations, including increased economic burden, a prolonged learning curve, extended surgery time, and equipment dependency. Consequently, the application of hip arthroscopy for femoral neck fractures will necessitate further clinical studies to elucidate the clinical effects and complications.

In conclusion, this study reports for the first time of a minimally invasive reduction technique of using hip arthroscope to assist the reduction of irreducible subcapital femoral neck fracture. Large scale clinical trial will be performed to compare the clinical effectiveness, to identify the indications, contraindications in future.

## Data Availability

The original contributions presented in the study are included in the article/Supplementary Material, further inquiries can be directed to the corresponding authors.
