# Supplementary material for: Hip arthroscope-assisted percutaneous reduction and fixation of displaced subcapital femoral neck fracture
Source: Front Surg. 2025 Apr 1;12:1555752. doi: 10.3389/fsurg.2025.1555752 (PMC11996925; doi:10.3389/fsurg.2025.1555752)
Supplement: Supplementary file 1 [file Table1.docx]

**Table S1. Evaluations of patient reported outcomes at 1 month after surgery.**

| VAS |  | EQ-5D-3L |  | HHS |  | Oxford hip score |
| --- | --- | --- | --- | --- | --- | --- |
| 2 |  | 65 |  | 63 |  | 13 |

**Figure S1**


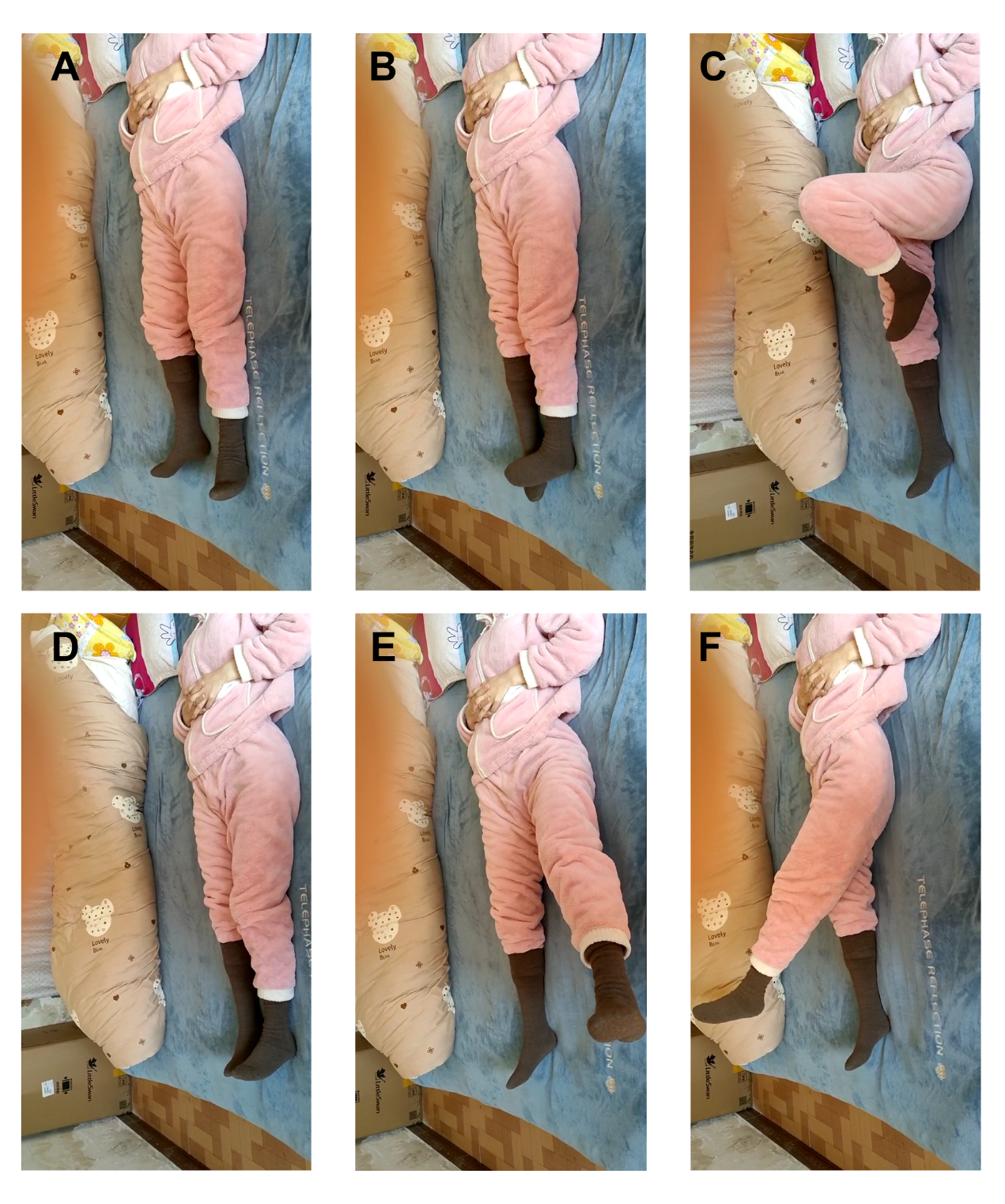


**Figure S1. Range of motion at 1 month after surgery.**

(A-B) External and internal rotation; (C-D) flexion and extension; (E-F) abduction and adduction at 1 month after surgery.
